# Flexible Ureteroscopic Management of Horseshoe Kidney Renal Calculi

**DOI:** 10.1590/S1677-5538.IBJU.2014.0086

**Published:** 2015

**Authors:** Jie Ding, Yunteng Huang, Siping Gu, Yifan Chen, Jie Peng, Qiang Bai, Min Ye, Jun Qi

**Affiliations:** 1 Department of Urology, Xin Hua Hospital Affiliated to Shanghai Jiao Tong University School of Medicine, Shanghai, China; 2 Micro-Invasive Surgery Center, Shishi Overseas Chinese Hospital, Fujian, China; 3 Department of Ophthalmology, Xin Hua Hospital Affiliated to Shanghai Jiao Tong University School of Medicine, Shanghai, China

**Keywords:** Horseshoe Crabs, Kidney, Urolithiasis, Ureteroscopes, Lasers, Solid-State

## Abstract

**Purpose:**

To evaluate the clinical efficacy of flexible ureteroscope (F-URS) combined with holmium laser lithotripter in treating renal calculi in horseshoe kidney.

**Materials and Methods:**

From November 2010 to December 2013, the medical history and charts of sixteen patients (mean age 42.9±11.6 years, range 26-66 years), including 13 males and 3 females were analyzed retrospectively. Mean stone burden was 29±8 mm (range 17-42 mm^2^). Mean stone digitized surface area (DSA) was 321±94 mm^2^ (range 180-538 mm^2^). Under spinal anesthesia in a modified lithotomy position with the head down, rigid ureteroscope was placed firstly into the ureter to reach the level of the pelvis, a zebra guide wire was inserted and following the removal of the rigid ureteroscope, an ureteral access sheath was positioned along the guide wire, then passed the URF P-5 flexible ureteroscope into the renal cavities over the guidewire. After locating the stones, holmium laser lithotripsy was performed.

**Results:**

The average operative time was 92±16 minutes (range 74-127 min.). No major complications were encountered. Ten patients obtained stone-free status with one session, four obtained stone-free status after two sessions. Single session stone-free rate was 62.5%, overall stone-free rate was 87.5%. Two patients have small residual stones in the lower pole.

**Conclusions:**

F-URS combined with holmium laser lithotripter and nitinol basket, is safe and effective in dealing with moderate stone diameter (<30 mm) in HSKs with high clearance rates and low complication rates.

## INTRODUCTION

The horseshoe kidney (HSK) is the most common renal fusion anomaly, with a prevalence of 0.25% of the population. During embryogenesis, the normal ascent of the kidneys is arrested by the fusion of the lower poles, resulting in malrotation with anterior displacement of the collecting system. Insertion of the ureter onto the renal pelvis is superiorly and laterally displaced. Impaired drainage of the collecting system and associating ureteropelvic obstruction predispose the patient to urinary tract infection (UTI) and urolithiasis. The latter of which is the most common complication of horseshoe kidney with an incidence of 21% to 60% (1).

On the other hand, a high incidence of metabolic abnormalities was identified in the stone-bearing patients with HSKs, suggesting that metabolic abnormalities, rather than anatomical anomaly or urinary stasis, contribute largely to the propensity of urolithiasis in HSKs (2–5).

Once seen as standard treatment of HSK urolithiasis, open surgery is now practically obsolete. With the advent and development of minimally invasive instruments, extracorporeal shock wave lithotripsy (SWL) and percutaneous nephrolithotomy (PNL) are currently the most commonly applied strategies for treating calculi in HSKs. Small (<15 mm) and uncomplicated calculi could be treated conveniently by SWL (6–9). However, for larger ones, the stone-free rate of SWL is much less than that of PNL because malformed anatomic structures often failed to provide spontaneous and complete drainage of stone fragments. Since the 1980s, following a host of successful reports (10–13), PNL has been widely adopted as the standard treatment for HSK stone diseases if SWL fails or the stone is greater than 20 mm in diameter (1, 14).

With the advancement of technology, small actively-deflectable flexible ureteroscopy (F-URS), equipped with holmium laser lithotripter and nitinol baskets or graspers, has stood out to be a promising alternative harboring similar effectiveness and stone-free rates. Although an increasing number of cases on retrograde management of renal calculi in patients with HSK have been reported (15–18), the total number is still rather limited and reported mean stone diameter is small (around 15 mm), so we share our single center experience of utilizing F-URS in treating moderate stone burden in HSKs, and comparatively review results from other reports, hoping to provide more information for clinicians when they are considering alternative treatment modalities.

## MATERIALS AND METHODS

From November 2010 to December 2013, a total of 16 patients (mean age 42.9±11.6, range 26-66 years) including 13 males and 3 females with HSK underwent F-URS with the holmium laser to treat renal calculi in our hospital. The main symptom of all six patients was mild to moderate flank or abdominal pain. Three patients had gross hematuria. Two patients had a single stone in the pelvis, one patient had a single stone in the middle calyx, one patient had a single stone in the lower calyx, and the other 12 patients had multiple stones. Five patents had mild to moderate hydronephrosis. Six patients had undergone SWL, one patient had undergone SWL and PNL. All procedures were performed by two experienced urologists. The demographic data and medical information were obtained from their medical records and charts (Table-1).

**Table 1:** Demographic characteristics and surgical statistics.

Variable		Value
Age (y)		42.9±11.6 (26-66)
**Gender (n)**		
	Male	13
	Female	3
Body Mass Index (kg/m2)		23.7±1.9 (19.7-27.2)
**Initial presentation (n)**		
	Flank pain	13
	Hematuria	3
**Urologic history (n)**		
	SWL	7
	PNL	1
Number of stones per person		2.1 (1-4)
**Stone status (n)**		
	Single	4
	Multiple	12
**Stone location (n)**		
	Pelvic	2
	Mixed calyceal	9
	Mixed Pelvic calyceal	5
Mean stone burden (mm)		29±8 (17-42)
Mean stone surface area (mm^2^)		321±94 (180-538)
Number of session per person		1.4 (1-2)
Single session stone-free rate		62.5%
Overall stone-free rate		87.5%
Operative time (min)		92±16 (74-127)
Hospital stay (d)		0.8±0.9 (0-3)
**Stone composition (n)**		
	Calcium oxalate monohydrate	9
	Calcium oxalate dihydrate	5
	Uric acid	2

**Figure 1:**
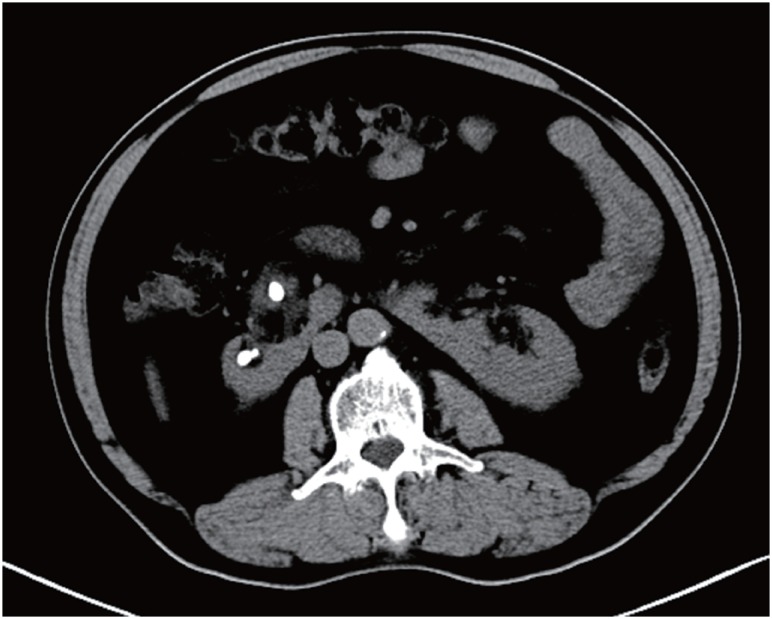
CT scan of renal stones in a horseshoe kidney.

Preoperative plain films, intravenous pyelography and CT scans of the urinary tract were reviewed to estimate the stone size and location and for evidence of possible structural obstructions (Figure-1). The largest stone was 26×18 mm. The average stone number was 2.1 (range 1-4). Stone burden is calculated by adding the largest diameter of different stones together as done in other reports, and mean stone burden was 29±8 mm (range 17-42 mm^2^) and mean digitized surface area (DSA) was 321±94 mm^2^ (range 180-538 mm^2^). No ureteropelvic junction obstruction was revealed, and no absolute contraindications were found. Although none had contraindications for PNL, such as morbid obesity, coagulation deficiency or severe cardiopulmonary diseases, all of them were scheduled to receive F-URS.

All procedures were performed with the patient under spinal anesthesia in a modified lithotomy position with the head slightly down. Firstly, an 8/9.8F Wolf rigid ureteroscope (Richard Wolf, Knittlingen, Germany) was introduced to visualize the ureteral orifice. After the rigid ureteroscope entered the ureter, a 0.038 inch Teflon guidewire was inserted. The ureteroscope went up to the junction of ureter and pelvis, and general distribution of renal calices was inspected, after which the ureteroscope was taken out. A 35 cm ureteral access sheath, 12/14F Flexor (Cook Urological, Spencer, IN) was introduced, then passed the URF P-3 or P-5 flexible ureteroscope (Olympus, Tokyo, Japan) into the renal cavities over the guidewire. After locating the stones, the 200μm holmium laser fiber (Lumenis GmbH, Dreieich, Germany) was introduced. Laser energy was maintained at 0.8-1.2 J, and frequency 10-15 Hz. Nitinol baskets were used to extract relatively large stone fragments for stone removal and component analysis, and attempts were made to relocate some stones from lower pole to suitable position. Once full fragmentation of the stones in sight was obtained and no fragment ?3 mm in size were left in sight, a F 6 double-J stent was placed and the guidewire removed. A F18 Foley catheter was left in place for one to three days to ensure maximal drainage. The patients were advised to drink enough water to facilitate evacuation of the small fragments by urine flow. Prophylactic antibiotics were administered intravenously on operative day and postoperative day 1 to prevent infections.

All patients were evaluated by plain films and ultrasound on postoperative day one. Four weeks later, patients returned to the hospital for follow-up, plain films taken, ultrasound detected and double-J stent removal.

## RESULTS

A total of 16 patients were included in our retrospective study. Plain films taken on postoperative day one and four weeks later showed that ten patients received only one session of F-URS and acquired stone-free status, and single-session stone-free rate was 62.5%; six patients received two sessions and then four of them acquired stone-free status, while the other two had small residual stone in the lower pole, 7×6 mm in one patient and 8×6 mm in the other. Lower calyceal stones were unable to be reached by the ureteroscope, and attempts for relocation by basket also failed. Overall stone free rate was 87.5% (14/16). The average operative time was 92±16 min. (range 74-127 min.). No major complications such as perforation of the ureter or sepsis were encountered, and no blood transfusion was needed. Three of our patients had moderate postoperative fever, and the fever all gradually subsided after adjusting antibiotics. Symptoms such as flank pain, hematuria were relieved in all of them, and hydronephrosis gradually resolved in all five patients. Postoperative follow-up last for 6-24 months, the patient with a 7×6 mm lower pole fragment remains the same, but the patient with a 8×6 mm fragment increased to 10×7 mm 21 months after surgery. One patient who was proven stone-free developed a 5×3 mm calculus in the middle calyx. Stone sample analysis was done for 15 patients. The main compositions of two were uric acid, nine were calcium oxalate monohydrate, and six were calcium oxalate dihydrate.

## DISCUSSION

Several relatively large and earlier studies had the conclusion that small stones in HSKs could be non-invasively managed by SWL with the average number of SWL treatment sessions per patient around 2, absolute stone-free rate of 50%-70%, while a significant proportion of patients required additional intervention including PNL, URS or open surgery (19–22). PNL gradually gained favor as a treatment for patients with large or medium sized renal calculi in HSKs regardless whether there was failed EWSL attempts or not. In earlier times, although stone-free rates of PNL were much higher, the abnormal anatomic structures of pelvis and calices, aberrant vasculature and retrorenal colon brought about a perception among many urologists that PNL in patients with HSKs is far more complicated and challenging compared with PNL in normal patients. However, more and more cheerful results have been reported, claiming the stone-free rate, number of access, major complication rate, length of hospital stay of PNL in patients with HSKs could be just as good as in that of PNL in normal kidneys (23–25). Undoubtedly, development of flexible nephroscopy contributed a lot to those data. Nevertheless, PNL on a HSK remains more difficult than on a normal kidney; blood transfusion are required in some of the patients and mean hospital stay is longer. For patients with advanced age, obesity, coagulopathy, cardiovascular or pulmonary disease, PNL is not an optional choice.

Just like in flexible nephroscopy, the advancement in flexible ureteroscopy has also improved its performance and durability, and extended its therapeutic indications (26, 27). Since 2005, case series reporting management of F-URS in HSKs began to emerge. Weizer et al. presented 8 cases of renal calculi treated by F-URS in anomalous kidneys, 4 of which were HSKs (17). The stone-free rate was 75%. Molimard et al. reviewed 17 patients with HSK who had undergone F-URS (18). After 1.5 sessions per patient, the stone-free rate was 88.2%. Atis et al. reported 25 HSK patients with a mean stone size of 17.8±4.5 mm. The stone-free rate was 70% after a single procedure (28).

Since 2010, after an increasing number of successful reports on using F-URS in treating HSK urolithiasis, we started to perform F-URS on HSK renal stone. In this retrospective study, our average stone DSA appears to be the largest among all published reports of treating renal stones in HSKs using F-URS, and we believe it will be increasingly easier to treat moderate stone burden with F-URS and generate better results as the technology further advances. In Molimard's earlier report, the mean DSA was 145±132 mm^2^ (range 24-500 mm2), while our mean DSA was 321 mm^2^, which suggests that, for stones <30 mm in diameter, F-URS with holmium laser lithotripsy could achieve similar clearance rate as small stones. Comparing with PNL, F-URS harbor advantages such as less invasiveness, no need for blood transfusion, shorter hospital stay and fewer contraindications.

As it is known to all, the maximum active deflection angle of a flexible ureteroscope decreases after the insertion of optical fiber or basket. Comparing with the previous generation of flexible ureteroscope, the upward/downward maximum active deflection angle of Olympus P-5 is 180º/270º respectively with a 90º field of view, capable of reaching any position in the kidney. In an actual experiment the maximum active deflection angle of Olympus P-5 is 180.4º/272.3º with a 200 μm Laser Vision optical fiber inside, 181.9º/280.6º with a 2.2 F Cook basket inside, almost the same comparing with empty state. However, the maximum active deflection radius significantly increases from 9.5 mm to 11.3 mm, 11.4 mm, which indicates that a larger space was required for the maximum deflection of the tip of the ureteroscope (29). It is worth mentioning that the pelvis of horseshoe kidney is flatter than that of normal kidney, the intrarenal space is narrower, which increase the difficulty of deflecting and steering the ureteroscope inside the kidney. Also, the abnormal structure of the kidneys, high insertion of the ureters and acute infundibulopelvic angle could negatively affect the stone-free rate and increase the risk of a second intervention (30). Atis et al. also found that stone size and location in lower pole were statistically significant factors for clearance failure of RIRS in HSK (28). In the operations of this study, we tried methods of modifying the position of the patients, relocating the lower pole calculi to upper or middle calyx and then initiate fragmentation, to improve clearance rate of lower pole stones. Relocation of lower pole calculi, on one hand prevents the stone fragments from escaping to unreachable position, on the other hand prevents the flexible ureteroscope from staying in the maximum deflective status, thus prolonging the usage of the ureteroscope and reducing repairing cost.

Because of the position and structure of the horseshoe kidney, the flexible ureteroscope needs to stay in large deflective status for relatively long time during the operation. So protection of the ureteroscope is of great importance. The usage of ureteral access sheath facilitates the insertion of the ureteroscope and passage of stone fragments, protects the shaft, and reduces the pelvic pressure during operation, thus reducing the incidence of postoperative fever or bacteremia. Since the normal ascent of kidneys is arrested, the position of HSK is lower than normal with the ureters also shorter, so we do not introduce the ureteral access sheath too deep. If the ureteral access sheath is inserted as deep as in a normal person, the tip might already pierce the mucosa of pelvis and cause bleeding. Careful preoperative radiologic study could provide urologists with more detailed information on the malformation of kidney and ureters, the position of calculi and calices. For patients with relatively large and hard stones, the urologists need to stop before the operation gets too long, and plan for a second session afterwards, to reduce the risk of postoperative infection.

Since the insertion of the ureter onto the renal pelvis is superiorly and laterally displaced, we propose a hypothesis that the impact of small stones in the lower calices in HSKs is lower than those stones in normal kidneys for it is more difficult for small stones to move to the ureteropelvic junction. Considering the low stone-free rate of SWL, we didn't advise the two patients with residual lower pole stones to take SWL treatment. Instead regular follow-up of ultrasound and KUB was encouraged. We suggest that once acute symptoms resolved, patients with small lower calyceal residual stones go on regular monitoring, and wait to receive other treatment modality such as PNL if inevitably required in the future. Patients with stone disease should also undergo metabolic evaluation and adjust their diet accordingly to minimize the risk of stone recurrence since metabolic abnormalities contribute to stone formation greatly, and proper medical therapy might aid in the future (31).

As this report is a retrospective analysis of our single center experience of flexible ureteroscopy management of horseshoe kidney renal calculi done by two surgeons with a single type of endoscope, the case number is still limited. What's more, due to financial reasons and fear of radiation impact of CT, most patients refused to receive CT scans after operation, so this might present the stone-free rate higher than the fact, especially in cases of radiolucent stones, if only plain films and ultrasound are used. More cases from multicenter performed with different types of state-of-the-art ureteroscope by different endourologists are required to validate and update the efficacy of flexible ureteoscope management of horseshoe kidney urolithiasis, and long-term follow-up comparison of calculi recurrence with CT between patients who are stone-free and patients with residual stones were also needed to confirm our hypothesis.

## CONCLUSIONS

Although anatomic anomaly might render stones in the lower calices difficult to be evacuated and affect the stone-free rate, our experience suggests that flexible ureteroscope, together with holmium laser lithotripter, remains effective in dealing with moderate stone size (<30 mm) in HSKs. As the technology further develops, F-URS will certainly contribute more and more to the non-invasive treatment for stones in abnormal kidneys.
